# Phytochemicals from *Leucas zeylanica* Targeting Main Protease of SARS-CoV-2: Chemical Profiles, Molecular Docking, and Molecular Dynamics Simulations

**DOI:** 10.3390/biology10080789

**Published:** 2021-08-17

**Authors:** Mycal Dutta, Abu Montakim Tareq, Ahmed Rakib, Shafi Mahmud, Saad Ahmed Sami, Jewel Mallick, Mohammad Nazmul Islam, Mohuya Majumder, Md. Zia Uddin, Abdullah Alsubaie, Abdulraheem S. A. Almalki, Mayeen Uddin Khandaker, D.A. Bradley, Md. Sohel Rana, Talha Bin Emran

**Affiliations:** 1 Department of Pharmacy, BGC Trust University Bangladesh, Chittagong 4381, Bangladesh; mycal@bgctub.ac.bd (M.D.); jewel@bgctub.ac.bd (J.M.); zia@bgctub.ac.bd (M.Z.U.); 2 Department of Pharmacy, Jahangirnagar University, Savar, Dhaka 1342, Bangladesh; 3 Department of Pharmacy, International Islamic University Chittagong, Chittagong 4318, Bangladesh; montakim0.abu@gmail.com (A.M.T.); sayeadiiuc@gmail.com (M.N.I.); 4 Department of Pharmacy, Faculty of Biological Sciences, University of Chittagong, Chittagong 4331, Bangladesh; rakib.pharmacy.cu@gmail.com (A.R.); s.a.sami18pharm@gmail.com (S.A.S.); 5 Microbiology Laboratory, Department of Genetic Engineering and Biotechnology, University of Rajshahi, Rajshahi 6205, Bangladesh; shafimahmudfz@gmail.com; 6 Drug Discovery, GUSTO A Research Group, Chittagong 4203, Bangladesh; mohuyamajumderbgctub@gmail.com; 7 Department of Physics, College of Khurma, Taif University, Taif 21944, Saudi Arabia; a.alsubaie@tu.edu.sa; 8 Department of Chemistry, Faculty of Science, Taif University, Taif 21974, Saudi Arabia; almalki.a@tu.edu.sa; 9 Centre for Applied Physics and Radiation Technologies, School of Engineering and Technology, Sunway University, Bandar Sunway 47500, Malaysia; mayeenk@sunway.edu.my (M.U.K.); d.a.bradley@surrey.ac.uk (D.A.B.); 10 Department of Physics, University of Surrey, Guilford GU2 7XH, UK

**Keywords:** COVID-19, SARS-CoV-2, main protease, *Leucas zeylanica*, GC-MS, molecular dynamics simulation

## Abstract

**Simple Summary:**

Molecular docking in conjunction with molecular dynamics simulation was accomplished as they extend an ample opportunity to screen plausible inhibitors of the main protease from *Leucas zeylanica*. The preferential phytochemicals were identified from *L. zeylanica* through gas chromatography–mass spectrometry (GC-MS). The pre-eminent three identified phytochemicals exhibited toxicity by no means during the scrutinization of ADME/T prominences. Moreover, pharmacologically distinguishing characteristics and the biological activity of the lead phytochemicals were satisfying as an antiviral drug contender. Additionally, the molecular dynamics simulation exhibited thermal stability and a stable binding affinity of the protein–compound complex that referred to the appreciable efficacy of lead optimization. Therefore, the preferable phytochemicals are worth substantial evaluation in the biological laboratory to recommend plausible antiviral drug candidates.

**Abstract:**

Severe acute respiratory syndrome coronavirus 2 (SARS-CoV-2), a contemporary coronavirus, has impacted global economic activity and has a high transmission rate. As a result of the virus’s severe medical effects, developing effective vaccinations is vital. Plant-derived metabolites have been discovered as potential SARS-CoV-2 inhibitors. The SARS-CoV-2 main protease (M^pro^) is a target for therapeutic research because of its highly conserved protein sequence. Gas chromatography–mass spectrometry (GC-MS) and molecular docking were used to screen 34 compounds identified from *Leucas zeylanica* for potential inhibitory activity against the SARS-CoV-2 M^pro^. In addition, prime molecular mechanics–generalized Born surface area (MM-GBSA) was used to screen the compound dataset using a molecular dynamics simulation. From molecular docking analysis, 26 compounds were capable of interaction with the SARS-CoV-2 M^pro^, while three compounds, namely 11-oxa-dispiro[4.0.4.1]undecan-1-ol (−5.755 kcal/mol), azetidin-2-one 3,3-dimethyl-4-(1-aminoethyl) (−5.39 kcal/mol), and lorazepam, 2TMS derivative (−5.246 kcal/mol), exhibited the highest docking scores. These three ligands were assessed by MM-GBSA, which revealed that they bind with the necessary M^pro^ amino acids in the catalytic groove to cause protein inhibition, including Ser144, Cys145, and His41. The molecular dynamics simulation confirmed the complex rigidity and stability of the docked ligand–M^pro^ complexes based on the analysis of mean radical variations, root-mean-square fluctuations, solvent-accessible surface area, radius of gyration, and hydrogen bond formation. The study of the postmolecular dynamics confirmation also confirmed that lorazepam, 11-oxa-dispiro[4.0.4.1]undecan-1-ol, and azetidin-2-one-3, 3-dimethyl-4-(1-aminoethyl) interact with similar M^pro^ binding pockets. The results of our computerized drug design approach may assist in the fight against SARS-CoV-2.

## 1. Introduction

In recent decades, the world has witnessed an unprecedented number of life-threatening human disease outbreaks caused by an array of pathogenic organisms, including several notable viral diseases, such as influenza, chikungunya, Nipah, Zika, and Ebola [1,2]. However, the ongoing spread of coronavirus disease 2019 (COVID-19) has been exponential. It has already surpassed most previous viral infections in terms of infectivity and has become the center of global attention. Wuhan, a populous Chinese city located in the Hubei province was the first location where this acute respiratory infection was identified in late December 2019 [3]. COVID-19 has taken a significant toll on people worldwide and on 11 March 2020 was declared by the World Health Organization (WHO) a pandemic. This highly contagious infection has had a detrimental impact on the global healthcare management system and, as of 1 February 2021, >100 million confirmed cases have been reported, including more than 2 million estimated deaths worldwide [4].

SARS-CoV-2 is a pleomorphic, enveloped, nonsegmented, single-stranded RNA beta-coronavirus belonging to the Coronaviridae family and features a large genome (27–32 kb) that encodes both structural and nonstructural proteins. SARS-CoV-2 is associated with a higher transmission rate than other well-known human beta-coronaviruses, such as SARS-CoV and Middle Eastern respiratory syndrome coronavirus (MERS-CoV) [5]. The coronavirus main protease (M^pro^) is a nonstructural protein that plays a crucial role in protein translation, viral replication, and maturation [6,7]. In a recent study, Liu et al. confirmed the existence of the M^pro^ (also known as 3CL^Pro^ or chymotrypsin-like protease) enzyme in SARS-CoV-2 [8]. The genome of SARS-CoV-2 encodes pp1a and pp1ab, two large polyproteins, similar to other Coronaviridae genomes [9]. The resulting polyproteins, pp1a and pp1ab, must be cleaved to generate mature nonstructural proteins (nsps) [10]. The large pp1a (replicase 1ab) is generated inside the cell via genomic RNA transcription. Therefore, the inhibition of M^pro^ activity is anticipated to result in the prevention of viral replication, as similar cleavage specificity has not been identified in any human proteases [11], indicating that the polypeptide cannot be properly cleaved in the absence of M^pro^. Additionally, M^pro^ has very low cytotoxicity and low similarity with human proteases [12]. Proteins produced by this pathogenic organism have been demonstrated to intervene the host immune response, and M^pro^ enzyme-specific T cells have been encountered in SARS-CoV-2 patients [13,14]. Therefore, M^pro^ is considered to represent a promising drug target for antiviral drug development.

In general, cytokine production, cell death, inflammation, and other pathophysiological processes are commonly associated with disruptions in redox balance, resulting in oxidative stress during viral infections, which can negatively affect the respiratory tract. Previously, viral replication was strongly correlated with the excessive production of reactive oxygen species (ROS) and a reduction in the components of antioxidant mechanisms [15,16,17,18]. Inflammatory reactions are triggered by the COVID-19 infection, resulting in the subsequent release of proinflammatory cytokines, which can cause acute lung damage [19]. Oxidative stress also plays a critical role in the perpetuation of the cytokine storm cycle and is important for blood clotting mechanisms [20]. The observed increase in COVID-19 infection severity in patients diagnosed with chronic diseases has been linked with the poor performance of the antioxidant system, suggesting that antioxidants may represent a prospective therapeutic option for COVID-19 infection [21].

A close connection exists between the innate immune response and the thrombotic response, and recent COVID-19 clinical data have revealed a correlation between this infection and thrombotic complications, which might result in increased incidence of microvascular thrombosis, venous thromboembolic illness, and stroke. Markers of COVID-19 include thrombotic complications, which are often associated with multiorgan failure and increased fatality [22].

Recently, phytochemicals have been investigated against different target proteins of SARS-CoV-2 to find appropriate lead compounds for COVID-19 infection. Baicalin, baicalein, 25-hydroxycholesterol, chrysosplenetin, shikonin, panduratin A, and quercetin are some of the examples of plant compounds that exhibited a potential effect against SARS-CoV-2 during in vitro studies. In silico studies were conducted on a broad spectrum for a plethora of medicinal plants and a huge number of compounds were screened. This not only helped indicate the potentiality of natural plant compounds but also reduced the number of tedious and costly wet-laboratory experiments [23]. Therefore, in our current study, we endeavored to assess the roles of phytoconstituents identified from *Leucas zeylanica,* a medicinal plant belonging to the Lamiaceae family, in the management of COVID-19 infection by employing computational biology approaches. Phytocompounds possess a wide range of pharmacological activities, and traditional healers have employed plants belonging to the *Leucas* genus to treat various disease states, indicating an immense potential for the discovery of new lead compounds [24]. *L. zeylanica* is a weed commonly referred to as “Ceylon slitwort” and locally known as “Kusha” [25]. The plant is widely distributed throughout China, India, Bangladesh, Sri Lanka, Thailand, Indonesia, Philippines, Vietnam, Cambodia, Nepal, Myanmar, Malaysia, and New Guinea [26]. A phytochemical screening of an *L. zeylanica* methanol extract confirmed the presence of alkaloids, flavonoids, tannins, steroids, and glycosides, which contribute to the traditional medicinal properties of the plant [27]. This plant is traditionally used as a vermifuge ingredient in addition to the treatment of burning sensations during urination, scabies, convulsion, fever, jaundice, scorpion and snake bites, colds, rheumatism, roundworm, psoriasis, anorexia, flatulence, colic, and malaria [24,25,28,29]. The antimalarial drugs chloroquine and hydroxychloroquine have been suggested as potential anti-COVID-19 therapies in recent studies; therefore, the traditional use of *L. zeylanica* against malaria may be significant [30]. The ethnopharmacological activities of this plant, including anti-inflammatory, antidiarrheal, antimicrobial, antioxidant, thrombolytic, hepatoprotective, analgesic, larvicidal, and insecticidal activities, have been reported in previous studies [25,26,31]. Importantly, the plant exhibits significant antioxidant and thrombolytic properties, which may be useful against these components of the SARS-CoV-2 pathogenesis. This study computationally investigated the roles played by compounds identified in *L. zeylanica* to combat SARS-CoV-2.

## 2. Materials and Methods

### 2.1. Collection and Identification of Plant Material

The aerial parts of *L. zeylanica* were collected from the forest area of Chittagong Hill Tract in November 2015, which was acknowledged by a prominent botanist from Bangladesh Forest Research Institute, Chittagong, Bangladesh. The Bangladesh National Herbarium has stored this plant for future reference with a voucher specimen for identification (accession no. BFRI-107).

### 2.2. Preparation of Plant Extract and Decoction Preparation

The aerial parts of the *L. zeylanica* plant were cleaned and cut into tiny pieces (0.4–0.5 mm), dried in air and ground (Moulinex Blender AK-241, Moulinex, France) into a powder (40–80 mesh, 355 g). The powder was immersed in 2 L of methanol for approximately 5 min, and the decoction was allowed to stand for 30 min before being filtered through Whatman No. 1 filter paper. At a temperature of less than 50 °C, filtrate from cheesecloth and Whatman filter paper No. 1 was condensed using a rotating evaporator (RE 200, Bibby Sterling Ltd., Staffordshire, UK). The extracts were placed into glass Petri dishes (90 × 15 mm, Pyrex, Germany) and allowed to air dry to evaporate the solvent completely, resulting in a final yield of 4.4%–5.6% *w*/*w*.

### 2.3. Gas Chromatography–Mass Spectrometry (GC-MS) Analysis

The aerial parts of *L. zeylanica* (methanol extract) were inspected with a mass spectrometer (TQ 8040, Shimadzu Corporation, Kyoto, Japan), using an electron-impact ionization process, combined with a gas chromatograph (GC-17A, Shimadzu Corporation), using a silica capillary (Rxi-5 ms; 0.25 m film, 30 m long and internal diameter 0.32 mm). The oven temperature was set at 70 °C (0 min); 10 °C, 150 °C (5 min); 12 °C, 200 °C (15 min); 12 °C, 220 °C (5 min), with a hold time of 10 min. The inlet temperature was 260 °C. A rate of 0.6 mL/min was used for the flux at a constant pressure of 90 kPa using helium gas. The temperature interface between the GC and MS was maintained at a constant 280 °C. The MS was performed in scan mode, with a range from 40 to 350 amu. The sample was injected at 1 µL, and the entire GC-MS process lasted for 50 min [32]. The results were compared against the National Institute of Standards and Technology (NIST) GC-MS library version 08-S for the identification of compounds in the peak areas.

### 2.4. Molecular Docking Study

#### 2.4.1. Ligand Preparation

From the GC-MS analysis, 34 compounds (a total of 35 with the standard) were downloaded in *.sdf* two-dimensional (2D) format from the PubChem database (https:/pubchem.ncbi.nlm.nih.gov/, accessed on 12 March 2021). For the preparation of the ligand, the LigPrep tool (Maestro v 11.1) was used. The pH 7.0 ± 2.0 was used for the generation of ionization states of the compounds with Epik 2.2 (Force field: OPLS3) in Schrödinger ver.11.1. Up to 32 possible stereoisomers per ligand were retained.

#### 2.4.2. Protein Preparation

The three-dimensional (3D) structure of the SARS-CoV-2 M^pro^ (PDB ID: 6LU7) [33] was retrieved from the RCSB Protein Data Bank (https://www.rcsb.org/structure/, accessed on 12 March 2021) in PDB format [34]. The Protein Preparation Wizard (Schrödinger ver.11.1) was used to prepare the 6LU7 receptor using the following processes: optimization, removal of water molecules, and minimization (Force field: OPLS3).

#### 2.4.3. Receptor Grid Generation and Glide Molecular Docking

The grid generation (Schrödinger ver.11.1) for the selected receptor was performed using the default parameters (Force field: OPLS3). Receptor grids were calculated for the prepared proteins for the observation of poses by various ligands bound within the active predicted site during the docking procedure. The van der Waals radius scaling factor and the partial atomic charge were 1.00 and 0.25, respectively. A cubic box of specific dimensions centered on the centroid of the active site residues was obtained for the receptor. The bounding box was set to 14 × 14 × 14 Å for docking experiments. Ligand docking was followed by the flexible standard precision (Schrödinger ver.11.1), and the docking score and the interactions of the ligand docking were recorded [35]. The results were represented as negative scores in kcal/mol, the final scoring was done on energy-minimized poses and shown as a Glide score. Discovery Studio (DS) version 4.5 was utilized to generate the 2D and 3D representations of the compounds [36]. The figures were generated using Microsoft PowerPoint 2019.

### 2.5. ADME/T Properties Analysis

The pharmacokinetic characteristics of all identified phytocompounds were evaluated and screened for drug candidacy using Lipinski’s rule of five (RO5) [37]. According to Lipinski’s RO5, a compound may exhibit optimal drug-like behavior if the selected parameters are within the specified limit and do not violate more than one of the following five criteria: molecular weight <500 g/mol; ≤5 hydrogen bond donors; ≤10 hydrogen bond acceptors; lipophilicity <5; and molar refractivity between 40 and 130. The web-based tool SwissADME, which is considered to be a convenient drug discovery tool, was used to analyze the drug-likeness criteria of the detected biological compounds [38]. Compounds that pass Lipinski’s RO5 can be considered suitable candidates for the development of new drug entities.

### 2.6. Molecular Dynamics Simulation

The dynamic motion of the drug–protein complex was evaluated through a molecular dynamics simulation study. The simulation study was conducted using the YASARA software (version 20.1.1) [39] with the aid of the AMBER14 force field [40]. The N3 inhibitor complex was used as a control system to be compared against the other three complexes. The periodic boundary condition was maintained, and a cubic simulation cell was created that was 20 Å larger than the biological systems in all cases. The NPT ensemble method was used and a Berendsen thermostat was applied to control the temperature of four systems. For the calculation of long-range electrostatic interactions, the particle mesh Ewald method [41] was applied, and the short-range van der Waals and Coulomb interactions were analyzed using a cutoff radius of 8 Å. The TIP3P or transferable intramolecular potential 3 points were used to add Na and Cl ions [42]. The total physiological conditions of the system were set to a temperature of 298 K, pH 7.4, and 0.9% NaCl. For the initial energy minimization process, the steepest descent gradient approach was used with a simulated annealing method. The molecular dynamics simulation trajectories were saved after every 100 ps using a time step of 1.25 fs [43]. The simulation study was conducted for 100 ns to analyze the root-mean-square deviation (RMSD), root-mean-square fluctuation (RMSF), radius of gyration (Rg), solvent-accessible surface area (SASA), secondary structure, and the number of hydrogen bonds [44,45,46]. The molecular mechanics Poisson–Boltzmann surface area (MM-PBSA) method was applied to calculate the binding free energy. The YASARA macro file was edited for this calculation [47,48]. The 1000 trajectory files were considered for MM-PBSA calculation.

## 3. Results

### 3.1. GC-MS Analysis

**Figure 1:**
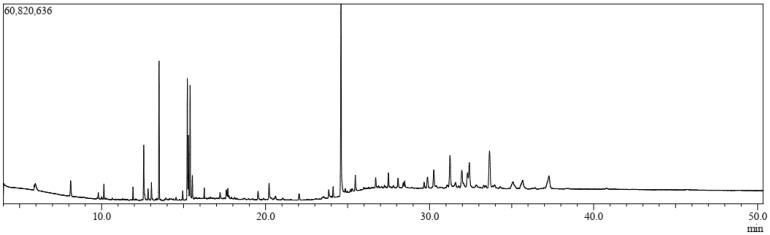
Total ionic chromatogram (TIC) for the methanolic extract of the aerial parts of *L. zeylanica* via gas chromatography–mass spectrometry (GC-MS).

A total of 34 compounds were identified from the aerial parts of *L. zeylanica* using GC-MS, which are listed in Figure 1 and Table 1, along with their chemical compositions. The total ionic chromatogram (TIC) is shown in Figure 1. Thirty-four compounds were selected for molecular docking analyses because the specific biological activities of interest for these compounds have not yet been established.

### 3.2. Molecular Docking Study

**Figure 2:**
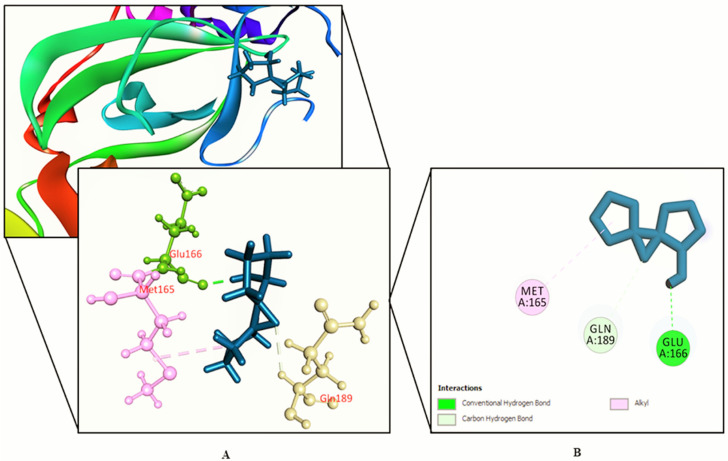
Shown here is 11-oxa-dispiro[4.0.4.1]undecan-1-ol binding the SARS-CoV-2 receptor (PDB ID: 6LU7). (**A**) A 3D representation and (**B**) 2D representation. Hydrogen bonds are displayed as green balls and sticks, hydrophobic bonds (Pi–Pi/Pi–sigma/amide–Pi interactions) are displayed as violet balls and sticks, hydrophobic bonds (Pi–alkyl/alkyl interactions stacking) are displayed as pink balls and sticks, hydrophobic bonds (Pi–sulfur) are displayed as gold balls and sticks, and carbon–hydrogen bonds are displayed as white balls and sticks.

**Figure 3:**
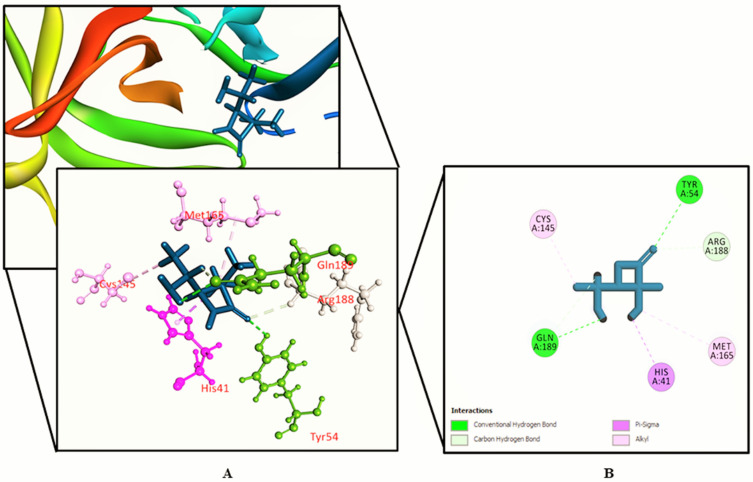
Azetidin-2-one 3,3-dimethyl-4-(1-aminoethyl) binding the SARS-CoV-2 receptor (PDB ID: 6LU7). (**A**) A 3D representation and (**B**) 2D representation. Hydrogen bonds are displayed as green balls and sticks, hydrophobic bonds (Pi–Pi/Pi–sigma/amide–Pi interactions) are displayed as violet balls and sticks, hydrophobic bonds (Pi–alkyl/alkyl interactions stacking) are displayed as pink balls and sticks, hydrophobic bonds (Pi–sulfur) are displayed as gold balls and sticks, and carbon–hydrogen bonds are displayed as white balls and sticks.

**Figure 4:**
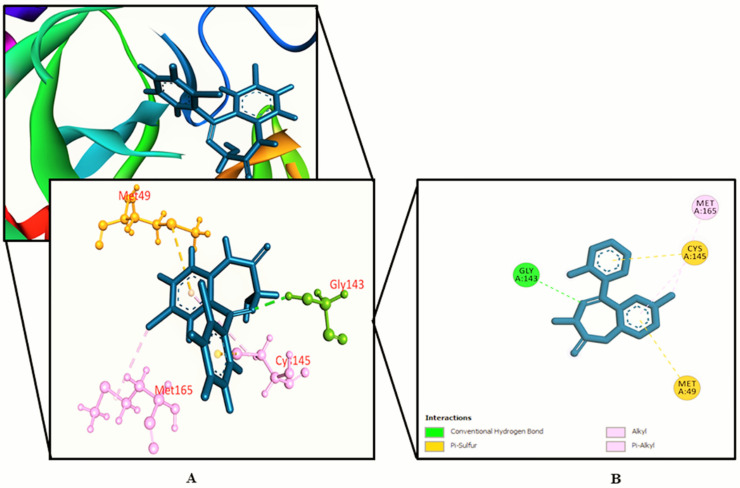
Lorazepam, 2TMS derivative binding the SARS-CoV-2 receptor (PDB ID: 6LU7). (**A**) A 3D representation and (**B**) 2D representation. Hydrogen bonds are displayed as green balls and sticks, hydrophobic bonds (Pi–Pi/Pi–sigma/amide–Pi interactions) are displayed as violet balls and sticks, hydrophobic bonds (Pi–alkyl/alkyl interactions stacking) are displayed as pink balls and sticks, hydrophobic bonds (Pi–sulfur) are displayed as gold balls and sticks, and carbon–hydrogen bonds are displayed as white balls and sticks.

**Figure 5:**
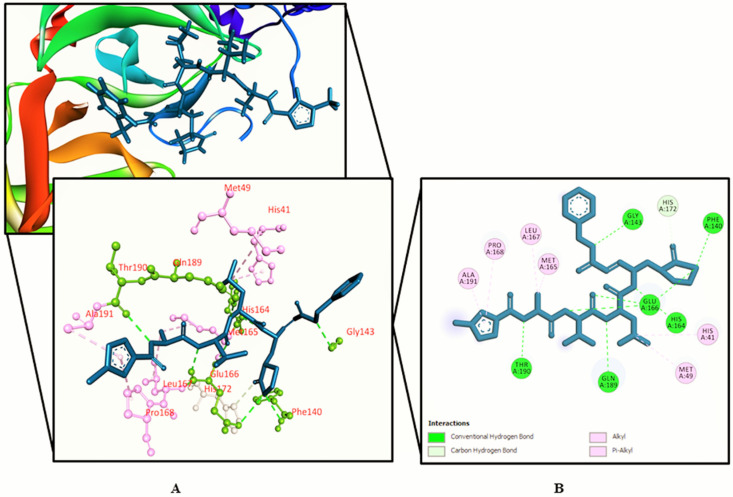
Standard inhibitor N3 binding the SARS-CoV-2 receptor (PDB ID: 6LU7). (**A**) A 3D representation and (**B**) 2D representation. Hydrogen bonds are displayed as green balls and sticks, hydrophobic bonds (Pi–Pi/Pi–sigma/amide–Pi interactions) are displayed as violet balls and sticks, hydrophobic bonds (Pi–alkyl/alkyl interactions stacking) are displayed as pink balls and sticks, hydrophobic bonds (Pi–sulfur) are displayed as gold balls and sticks, and carbon–hydrogen bonds are displayed as white balls and sticks.

The interactions between various identified compounds from *L. zeylanica* and the SARS-CoV-2 receptor (PDB ID: 6LU7) are presented in Table 2. Of the 34 compounds analyzed, 26 compounds interacted with the SARS-CoV-2 receptor, and the three compounds with the highest docking scores were identified as 11-oxa-dispiro[4.0.4.1]undecan-1-ol (−5.755 kcal/mol), azetidin-2-one 3,3-dimethyl-4-(1-aminoethyl) (−5.39 kcal/mol), and lorazepam, 2TMS derivative (−5.246 kcal/mol), as represented in Figure 2, Figure 3, Figure 4 and Figure 5 and Supplementary Figure S1. Importantly, the docking experiment delineated that standard inhibitor N3 showed the highest docking score (−7.013 kcal/mol) for the SARS-CoV-2 M^pro^ compared to the other studied compounds.

### 3.3. ADME/T Properties Analysis

Lipinski’s rules of five was employed to evaluate the various pharmacokinetic parameters of the identified phytocompounds. Eleven phytocompounds, namely hexadecamethylcyclooctasiloxane, octadecamethylcyclononasiloxane, cyclodecasiloxane, eicosamethyl, *N*,*N*′-methylenebis(oleamide), (Z,Z); squalene, α-tocopheryl acetate, bis(heptamethylcyclotetrasiloxy)hexamethyltrisiloxane, stigmasterol, γ-sitosterol, 9,19-cyclolanost-24-en-3-ol, acetate, (3.beta.), and stigmast-4-en-3-one failed to fulfill Lipinski’s RO5, as these compounds contravened more than one rule. However, 23 other compounds conformed with Lipinski’s RO5 and may demonstrate optimal drug-like behavior. The result of the absorption, distribution, metabolism, and excretion/toxicity (ADME/T) analysis is shown in Table 3.

### 3.4. Molecular Dynamics Simulation 

**Figure 6:**
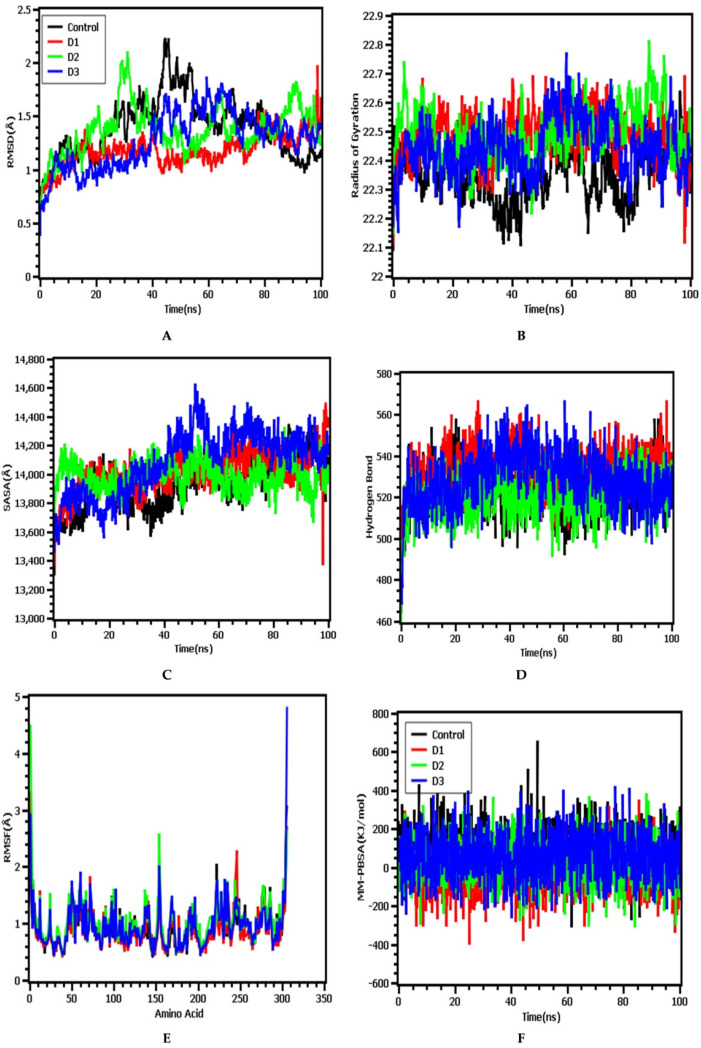
The molecular dynamics simulation of the four docked complexes. (**A**–**F**) Present, RMSD, Rg, SASA, hydrogen bonds, RMSF, and MM-PBSA analyses, respectively. Here, lorazepam, 11-oxa-dispiro[4.0.4.1]undecan-1-ol, azetidin-2-one-3, 3-dimethyl-4-(1-aminoethyl), and inhibitor N3 were denoted as D1, D2, D3, and control, respectively.

In this simulation study, lorazepam, 11-oxa-dispiro[4.0.4.1]undecan-1-ol, azetidin-2-one-3, 3-dimethyl-4-(1-aminoethyl), and inhibitor N3 were denoted as D1, D2, D3, and control, respectively. The RMSD from Figure 6A illustrated that the control drug–protein complex had a higher RMSD trend compared with those of the other three complexes. Initially, none of the four complexes displayed large fluctuations, and they generally remained in a steady state. However, after 40 ns, all complexes had slightly higher and lower RMSD trends, which indicated complex flexibility. Eventually, the complexes returned to a steady trend again for the remainder of the simulation time, exhibiting rigid profiles. The degree of mobility in a biological system can be indicated by the Rg profile. Figure 6B shows that the control drug–protein complex had a lower Rg profile than the experimental drugs, indicating the compacted nature of the protein complex, whereas higher Rg values, which correlate with the repeated folding and unfolding protein behavior, were observed for the protein complexes containing both D1 and D2. By contrast, the complex containing D3 demonstrated initial stability until 40 ns, after which the Rg value increased, which may represent the loose packaging of the system. However, after 60 ns the D3 complex regained a steady Rg pattern, similar to those observed for the other complexes.

The surface area of the biological systems and their corresponding binding patterns with ligand molecules can be assessed through SASA analysis. The D3 complex showed an increasing SASA value until 40 ns, after which the SASA stabilized (Figure 6C). This increasing trend in SASA represents protein expansion and comparatively loose binding. By contrast, D1, D2, and the control complex presented with stable SASA profiles and did not deviate. Therefore, these complexes experienced no changes in the surface area and formed more rigid profiles compared with the D3 complex. The quantitative measurement of hydrogen bonds in the drug–protein complex represents the constant nature and molecular recognition of the complexes. The D1 and D3 complexes formed more hydrogen bonds than the control and D2 complexes. More hydrogen bonds indicate an increasingly stable nature for the complex (Figure 6D).

The flexibility across the amino acid residues of the protein–drug system can be evaluated through RMSF descriptors. All of the complexes and their respective amino acid residues had lower RMSF values, indicating reduced flexibility. However, some residues, such as Ser1 (helix-strand), Gly2 (helix-strand), Leu50 (beta-turn), Arg60 (helix-strand), Asn72 (beta-turn), Lys97 (beta-turn), Tyr154 (beta-turn), Phe223 (beta-turn), His246 (helix-strand), Ser301 (beta-turn), Gly302 (beta-turn), Val303 (beta-turn), Thr304 (beta-turn), Phe305 (beta-turn), and Gln306 (beta-turn), presented with more flexibility associated with higher RMSF profiles in the molecular dynamics simulation (Figure 6E).

In molecular modeling and computational drug design schemes, the PBSA system is a widely used solvation model for the estimation of the binding free energy of the drug–protein complex. Better binding and more compact interactions are indicated by higher binding energy values in the MM-PBSA calculations [44,45,46]. The reference control protein structure of the protein had more binding energy, which indicated a positive interaction pattern. The complex molecules D2 and D3 had similar binding energy patterns to that observed for the control complex, whereas D1 had slightly less binding energy than the control complex, as demonstrated in Figure 6F.

## 4. Discussion

With an increasing number of cases worldwide, the COVID-19 situation continues to worsen on a daily basis, especially in developed countries such as the USA. The contagiousness of SARS-CoV-2 is above and beyond that of SARS-CoV or MERS-CoV, two other members of the beta-coronavirus family. However, no specific treatment has been developed to treat this infectious disease thus far. As a result, the mortality rate continues to increase rapidly. Due to the absence of specific therapeutic drugs, current treatment approaches primarily involve symptom relief and supportive care. Several clinical trials have been conducted for several drug candidates, including remdesivir, hydroxychloroquine, and lopinavir/ritonavir [49]. However, these drugs have not yet amassed sufficient evidence to support their clinical applications. Scientists and researchers worldwide are working together to identify treatment strategies for this deadly coronavirus. Recently, a research study suggested a role for immunopathological considerations in the treatment of SARS-CoV-2 [50]. Wang et al. showed that human monoclonal antibodies could have a neutralizing effect against SARS-CoV-2, primarily by targeting the spike glycoprotein of the virus [51]. However, traditional drug development processes are tedious, and the development of suitable drug candidates can take as long as 15 years, which is not a feasible approach for identifying appropriate cures for COVID-19. Computer-aided drug design may represent a potential method for identifying lead compounds to treat SARS-CoV-2, which can result in both rapid and accurate results. A plethora of studies that have applied computational biology techniques have successfully predicted novel lead compounds for combating SARS-CoV-2 in addition to designing in silico epitope-based vaccines against SARS-CoV-2 [2,5,30,52,53]. However, these types of studies continue to require further wet-lab verification to be developed into effective therapeutic strategies for COVID-19.

Medicinal plants have been used as an ideal source of therapeutic agents for thousands of years [54,55]. These therapeutic agents can be developed into medicines and can often be extracted from the crude extracts of several plant parts, including the leaves, stems, bark, rhizomes, fruits, and whole-plant materials [56,57]. The plant kingdoms feature several biomolecules that possess numerous and varied biological activities [58,59]. A recent review from Yang et al. reported a role for traditional medicinal practices in the management of SARS-CoV-2 infection [60]. However, due to the lack of comprehensive studies, the effects of biologically active molecules of plant origins against COVID-19 remain relatively unexplored. Through computational studies, researchers can predict the activities of several phytocompounds that might be effective for curbing the activity of the SARS-CoV-2 M^pro^ [30,61]. The SARS-CoV-2 M^pro^ plays important roles in viral replication and enzymatic cleavage, including the processing of the pp1a and pp1ab polyproteins. We have designed in silico studies to examine the inhibition of SARS-CoV-2 M^pro^ activity using phytocompounds identified from the methanolic extract of the aerial parts of *L. zeylanica*. The early detection of SARS-CoV-2 infections by the human immune system occurs through pattern recognition receptors (PRRs), which trigger a nuclear factor kappa B (NF-κB) response [62]. NF-κB activation regulates the inflammatory response through the overproduction of proinflammatory cytokines and chemokines. NF-κB has been considered to serve as a potential biomarker for oxidative stress, and the activation of NF-κB can be regulated by antioxidants [63]. Moreover, NF-κB activation leads to tissue factor expression, which ultimately induces a hypercoagulable state. Several previous studies have reported the antioxidant and thrombolytic attributes of ethanolic extracts of *L. zeylanica* [64,65]. Therefore, in the present study, we rationalize that several identified phytochemicals derived from the aerial parts of *L. zeylanica* could potentially play roles against SARS-CoV-2 M^pro^ activation.

Molecular docking is the most common computer-aided technique, which has been widely used during the last century [66]. Different algorithms have been developed to increase the accuracy of molecular docking analyses [67]. Molecular docking studies simulate the characteristics of small molecules during binding with the active site of a particular receptor protein. In the current study, we docked several identified phytochemicals identified in the aerial parts of *L. zeylanica* with the SARS-CoV-2 M^pro^ (PDB ID: 6LU7). In addition, to validate the molecular docking approach, we performed molecular docking analysis using a known molecule inhibitor, N3 (positive control), which was bound to the SARS-CoV-2 M^pro^ crystal structure (PDB ID: 6LU7). Although lorazepam, 2TMS derivative; azetidin-2-one 3, 3-dimethyl-4-(1-aminoethyl); and 11-oxa-dispiro[4.0.4.1]undecan-1-ol possessed the highest docking scores among the targeted compounds, their binding affinities were all weaker than that of the positive control N3. However, unlike inhibitor N3, the lorazepam, 2TMS derivative interacted with the Cys145 residue through both pi–alkyl and pi–sulfur interactions. Moreover, 3-butynoic acid; undecanoic acid, 11-bromo; methyl ester, 6-octadecenoic acid; methyl ester, (Z); hexadecanoic acid-2-hydroxy-1-(hydroxymethyl)ethyl ester; octadecanoic acid-2,3-dihydroxypropyl ester; 13-docosenamide, (Z); α-tocopheryl acetate; campesterol; stigmasterol; γ-sitosterol; 4-campestene-3-one-9; 19-cyclolanost-24-en-3-ol, acetate, (3-beta.); and stigmast-4-en-3-one also interacted with the Cys145 residue, and octadecanoic acid-2, 3-dihydroxypropyl ester; 13-docosenamide, (Z); and stigmasterol all interacted with Cys145 through the formation of a hydrogen bond. Additionally, azetidin-2-one 3, 3-dimethyl-4-(1-aminoethyl) and eight other compounds formed hydrophobic interactions with the His41 residue. The Cys145 and His41 residues have recently been identified as components of the SARS-CoV-2 M^pro^ catalytic dyads. 3-Butynoic acid and octadecanoic acid, 2,3-dihydroxypropyl ester each interacted with both the Gly143 and Ser144 residues through the formation of hydrogen bonds. Lorazepam, 2TMS derivative, undecanoic acid, 11-bromo- methyl ester, 6-octadecenoic acid, and methyl ester, (Z) also interacted with Gly143, whereas stigmasterol, 9,19-cyclolanost-24-en-3-ol, acetate, (3-beta.), and 4,22-cholestadien-3-one interacted with Ser144 through hydrogen bonding. In addition, 3-butynoic acid, undecanoic acid, 11-bromo-methyl ester, 3,7,11,15-tetramethyl-2-hexadecen-1-ol/phytol, 6-octadecenoic acid, methyl ester, (Z); octadecanoic acid, 2,3-dihydroxypropyl ester, 13-docosenamide, (Z); and inhibitor N3 all interacted with the His163 residue, but only octadecanoic acid, 2,3-dihydroxypropyl ester, and 13-docosenamide, (Z) formed hydrogen bonds with the His163 residue, whereas the remaining compounds, including inhibitor N3, yielded only hydrophobic interactions. Azetidin-2-one 3,3-dimethyl-4-(1-aminoethyl), 11-oxa-dispiro[4.0.4.1]undecan-1-ol, 3-azabicyclo[3.2.2]nonane, phytol acetate; phytol, hexadecanoic acid, methyl ester, 9,12-octadecadienoic acid (Z,Z), methyl ester, α-tocopheryl acetate; squalene, campesterol, γ-sitosterol; and 4-campestene-3-one interacted with the Met165 residue through hydrophobic interactions. The analysis of the docked compounds’ intermolecular interactions depicted the potentiality of the identified compounds’ ability to form interactions with the substrate binding cleft and essential residues in the active pocket of the SARS-CoV-2 M^pro^. The residues Gly143, Ser144, His163, His164, Met165, Glu166, Leu167, Asp187, Arg188, Gln189, Thr190, Ala191, and Gln192 have been indicated as playing pivotal roles by forming strong hydrogen bonds and substantial hydrophobic bond interactions with the SARS-CoV-2 M^pro^ [68,69]. Therefore, the predicted interactions of our identified plant compounds delineated as potential proteolytic function inhibitors of the SARS-CoV-2 M^pro^. Previous work has demonstrated both the antiviral and antioxidative properties of γ-sitosterol [70]. In addition, stigmasterol and its synthetic derivatives have been demonstrated to possess antiviral attributes [71,72]. Furthermore, a study by Okoro et al. showed that stigmasterol and bauerenol significantly inhibited the HIV-I integrase [73].

Although drug research has indicated a high level of interest in the investigation of natural products, realistically, the synthesis and purification of vast arrays of new compounds represent a research bottleneck [74]. To compensate for the time-consuming and costly nature of new product development, high-throughput screening techniques have been developed as effective methods for the identification of new hit compounds [75]. However, these hit compounds often result in pharmacokinetic failures that result in elimination following Phase II clinical trials [76]. Therefore, current research works have begun to involve key investigations of pharmacokinetics and pharmacodynamics parameters, which are often referred to as drug-likeness properties [77]. Several rules have been established to facilitate the acceptance of promising molecules, and Lipinski’s RO5 is the most commonly applied set of drug-likeness attributes [78]. In the current study, we analyzed the drug-likeness properties of the identified compounds based on Lipinski’s RO5 by utilizing the SWISS-ADME server. Our analysis indicated that most of the identified phytocompounds derived from the aerial parts of *L. zeylanica* followed Lipinski’s RO5. Furthermore, it was reported that, the antiviral drugs approved by the FDA in the last five years involve a molecular weight of 513.97, hydrogen bond donors of 2.95, and hydrogen bond acceptors of 9.13 [23]. The targeted natural compounds are also within the reported criteria, indicating that these compounds may be evaluated as potential drug molecules.

## 5. Conclusions

We used a methodology for computer-aided drug design to detect potent SARS-CoV-2 M^pro^ inhibitors following the *L. zeylanica* phytochemical analyses. Our research showed that several of the identified phytocompounds from *L. zeylanica* followed Lipinski’s RO5 and were therefore assessed as promising therapeutic molecules. The catalytic residues M^pro^, Ser 144, Cys 145, and His41 were shown to be linked in postmolecular dynamic structures to the investigated phytochemicals. The molecular dynamics simulations conducted for the docked complexes revealed more insight into the rigidity and binding stability of these protein–drug complexes. Following additional investigations in biological laboratories, these compounds may possibly be developed into effective SARS-CoV-2 therapeutic candidates.

## Figures and Tables

**Table 1:** List of compounds identified from the *L. zeylanica* methanolic extract using gas chromatography–mass spectrometry analysis.

Sl.	Name	RT	*m*/*z*	Area	Conc.	Peak Area (%)
1.	3-Butynoic acid	5.927	40.00	36,135	0.049	0.049299
2.	Tetradecamethylcycloheptasiloxane	8.221	40.00	23,061	0.031	0.031462
3.	Trimethylsilyl 2,6-bis[(trimethylsilyl)oxy]benzoate	10.139	73.00	1,516,895	2.066	2.069501
4.	Bis(heptamethylcyclotetrasiloxy)hexamethyltrisiloxane	11.914	73.00	1,205,683	1.642	1.644915
5.	Lorazepam, 2TMS derivative	11.914	73.00	1,205,683	1.642	1.644915
6.	Cyanoacetic acid	10.669	40.00	49,231	0.067	0.067166
7.	Methyl 11-bromoundecanoate	11.078	40.00	45,430	0.062	0.06198
8.	Azetidin-2-one 3,3-dimethyl-4-(1-aminoethyl)	11.078	40.00	45,430	0.062	0.06198
9.	3-Azabicyclo[3.2.2]nonane	11.916	40.00	67,327	0.092	0.091854
10.	Phytol acetate	12.567	68.00	2,737,101	3.728	3.73423
11.	Hexadecanoic acid, methyl ester	13.502	74.00	14,101,161	19.207	19.23823
12.	11-Oxa-dispiro[4.0.4.1]undecan-1-ol	13.503	40.00	91,678	0.125	0.125076
13.	Hexadecamethylcyclooctasiloxane	14.946	73.00	789,772	1.076	1.077487
14.	9,12-Octadecadienoic acid (Z,Z)-, methyl ester	15.225	67.00	6,028,138	8.211	8.224195
15.	6-Octadecenoic acid, methyl ester, (Z)-	15.225	55.00	2,986,886	4.068	4.075012
16.	Phytol	15.393	71.00	11,551,440	15.734	15.75964
17.	Methyl stearate	15.530	74.00	2,412,152	3.286	3.290901
18.	Octadecamethylcyclononasiloxane	16.257	73.00	913,521	1.244	1.246318
19.	Pseduosarsasapogenin-5,20-dien	17.219	83.00	547,313	0.745	0.7467
20.	Cyclodecasiloxane, eicosamethyl-	19.540	73.00	929,809	1.266	1.268539
21.	Hexadecanoic acid, 2-hydroxy-1-(hydroxymethyl)ethyl ester	20.211	98.00	971,087	1.323	1.324855
22.	Octadecanoic acid, 2,3-dihydroxypropyl ester	23.851	43.00	356,272	0.485	0.486062
23.	13-Docosenamide, (Z)-	24.596	59.00	15,302,725	20.844	20.87752
24.	*N*,*N*′-methylenebis(oleamide), (Z,Z)-	24.595	207.00	247,529	0.337	0.337704
25.	Squalene	27.770	207.00	138,619	0.189	0.189118
26.	α-Tocopheryl acetate	28.073	207.00	203,341	0.277	0.277418
27.	Campesterol	29.656	207.00	201,572	0.275	0.275005
28.	Stigmasterol	30.546	207.00	156,066	0.213	0.212921
29.	γ-Sitosterol	31.581	207.00	165,310	0.225	0.225533
30.	4-Campestene-3-one	31.581	207.00	147,960	0.202	0.201862
31.	9, 19-Cyclolanost-24-en-3-ol, acetate, (3.beta.)	32.728	207.00	101,495	0.138	0.13847
32.	4,22-Cholestadien-3-one	32.728	207.00	101,495	0.138	0.13847
33.	Stigmast-4-en-3-one	33.655	124.00	4,766,323	6.492	6.502699
34.	1,2-Bis(trimethylsilyl)benzene	35.243	207.00	163,549	0.223	0.22313

**Table 2:** Docking results (kcal/mol)) estimated for the top 34 compounds.

SL. No.	Name	Docking Score	Interactions by H-Bond	Hydrophobic Bonds (Pi–Alkyl/Alkyl Interaction)	Hydrophobic Bonds (Pi–Pi/Pi–Sigma/ Amide–Pi Interaction)	Pi–Sulfur Interaction
1.	3-Butynoic acid	−1.08	Cys 145, Ser 144 (2), Gly 143	His 172, His 163	Phe 140	−
2.	Tetradecamethylcycloheptasiloxane	−	−	−	−	−
3.	Trimethylsilyl 2,6-bis[(trimethylsilyl)oxy]benzoate	−	−	−	−	−
4.	Bis(heptamethylcyclotetrasiloxy)hexamethyltrisiloxane	−	−	−	−	−
5.	**Lorazepam, 2TMS derivative**	**−5.246**	Gly 143	Cys 145	−	Cys 145, Met 49
6.	Cyanoacetic acid	−3.469	Lys 61 (2), Arg 60, Asp 48	−	−	−
7.	Methyl 11-bromoundecanoate	−1.792	Gly 143	His 163, Cys 145	−	−
8.	**Azetidin-2-one 3,3-dimethyl-4-(1-aminoethyl)**	**−5.39**	Gln 189, Tyr 54	Met 165 (2)	His 41	−
9.	3-Azabicyclo[3.2.2]nonane	−4.703	−	His 41 (2), Met 49, Met 165	−	−
10.	Phytol acetate	−3.357	Met 165, Glu 166	Met 165, Cys 145 (2), Leu 27, His 41, Met 49, Met 165	−	−
11.	Phytol	−1.565	Asn 142,	His 163, His 172, Met 165 (2), His 41, Met 49	−	−
12.	Hexadecanoic acid, methyl ester	+0.991	Asn 142	Met 165,	His 41	−
13.	**11-Oxa-dispiro[4.0.4.1]undecan-1-ol**	**−5.755**	Glu 166	Met 165	−	−
14.	Hexadecamethylcyclooctasiloxane	−	−	−	−	−
15.	9,12-Octadecadienoic acid (Z,Z)-, methyl ester	−1.111	Glu 166	Met 165, Leu 167	−	−
16.	6-Octadecenoic acid, methyl ester, (Z)-	−0.399	Ser 144, Gly 143	Cys 145, His 163	His 41	−
17.	Methyl stearate	−0.419	Gln 189	His 41, Leu 50, Met 49	−	−
18.	Octadecamethylcyclononasiloxane	−	−	−	−	−
19.	Pseduosarsasapogenin-5,20-dien	−	−	−	−	−
20.	Cyclodecasiloxane, eicosamethyl-	−	−	−	−	−
21.	Hexadecanoic acid, 2-hydroxy-1-(hydroxymethyl)ethyl ester	−4.152	Glu 166, Cys 145	−	−	−
22.	Octadecanoic acid, 2,3-dihydroxypropyl ester	−2.406	His 163, Gln 143 (2), Ser 144, Cys 145	−	−	−
23.	13-Docosenamide, (Z)-	−4.46	His 163, Cys 145	His 41, Met 49	−	−
24.	*N*,*N*’′methylenebis(oleamide), (Z, Z)-	−4.057	Phe 140	His 41	−	−
25.	Squalene	−3.609	−	Met 165 (2), Met 49, Cys 149, His 41	−	−
26.	α-Tocopheryl acetate	−4.871	−	Leu 167, Met 165 (2), Met 49, His 41, Leu 27, Cys 145	−	−
27.	Campesterol	−3.776	Thr 24	Met 49 (2), His 41 (2), Met 165 (2), Cys 145	−	−
28.	Stigmasterol	−4.194	Cys 145, Ser 144 (2)	Ala 191, Pro 168	−	−
29.	γ-Sitosterol	−4.854	Thr 26	Cys 145 (3), His 41 (2), Met 49, Met 165 (2), Leu 167, Pro 168	−	−
30.	4-Campestene-3-one	−3.934	−	Met 165, Met 49 (2), His 41 (2), Cys 145	−	−
31.	9, 19-Cyclolanost-24-en-3-ol, acetate, (3.beta.)	−3.105	Asn 142, Ser 144	Cys 145, Pro 168, Ala 191, Leu 50	−	−
32.	4,22-Cholestadien-3-one	−3.824	Ser 144	Ala 191,	−	−
33.	Stigmast-4-en-3-one	−3.696		His 41 (2), Cys 145, Leu 27	−	−
34.	1,2-Bis(trimethylsilyl)benzene	−	−	−	−	−
35.	**Standard (inhibitor N3)**	**−7.013**	Phe 140, Gly 143, His 164, Glu 166, Gln 189, Thr 190	His 41, Met 49, Met 165, Leu 167, Pro 168, Ala 191	−	−

N.B.: Bold text indicates best docking scores.

**Table 3:** Pharmacological profile of the 23 ligand molecules passing the Lipinski’s RO5, as derived from the SwissADME webserver.

Compounds	Molecular Weight ^a^ (g/mol)	Hydrogen Bond Acceptors ^b^	Hydrogen Bond Donors ^c^	MlogP ^d^	Molar Refractivity ^e^	No. of Lipinski Violations ^f^
	<500	≤10	≤5	<5	40–130	≤1
3-Butynoic acid	84.07	2	1	0.38	21.28	1
Tetradecamethylcycloheptasiloxane	519.08	7	0	−1.54	129.97	1
Trimethylsilyl 2,6-bis[(trimethylsilyl)oxy]benzoate	370.66	4	0	2.97	103.15	0
Lorazepam, 2TMS derivative	465.52	4	0	4.66	134.90	1
Hexadecanoic acid, 2-hydroxy-1-(hydroxymethyl)ethyl ester	330.50	4	2	3.18	97.06	0
Cyanoacetic acid	85.06	3	1	−0.96	18.06	1
Methyl 11-bromoundecanoate	279.21	2	0	3.56	68.95	0
Azetidin-2-one 3,3-dimethyl-4-(1-aminoethyl)-	142.20	2	2	0.72	43.01	0
3-Azabicyclo[3.2.2]nonane	125.21	1	1	1.83	43.06	0
Phytol, acetate	338.57	2	0	5.47	108.68	1
Hexadecanoic acid, methyl ester	270.45	2	0	4.44	85.12	0
11-Oxa-dispiro[4.0.4.1]undecan-1-ol	168.23	2	1	1.52	46.16	0
9,12-Octadecadienoic acid (Z,Z)-, methyl ester	294.47	2	0	4.70	93.78	0
6-Octadecenoic acid, methyl ester, (Z)-	296.49	2	0	4.80	94.26	0
Phytol	296.53	1	1	5.25	98.94	1
Methyl stearate	298.50	2	0	4.91	94.73	0
Pseduosarsasapogenin-5,20-dien	414.62	3	2	4.42	123.27	0
Octadecanoic acid, 2,3-dihydroxypropyl ester	358.56	4	2	3.63	106.67	0
13-Docosenamide, (Z)-	337.58	1	1	5.06	110.30	1
Campesterol	400.68	1	1	6.54	128.42	1
4-Campestene-3-one	398.66	1	0	6.43	127.46	1
4,22-Cholestadien-3-one	382.62	1	0	6.13	122.18	1
1,2-Bis(trimethylsilyl)benzene	222.47	0	0	4.13	72.40	0

## Data Availability

Available data are presented in the manuscript.

## Supplementary Materials

The following are available online at https://www.mdpi.com/article/10.3390/biology10080789/s1, Supplementary File S1: A 2D representation of 23 compounds against the SARS-CoV-2 receptor (PDB: 6LU7). (a) 3-Butynoic acid; (b) cyanoacetic acid; (c) methyl 11-bromoundecanoate; (d) 3-azabicyclo[3.2.2]nonane; (e) phytol acetate; (f) 3,7,11,15-tetramethyl-2-hexadecen-1-ol; (g) hexadecanoic acid, methyl ester; (h) 9,12-octadecadienoic acid (Z,Z)-, methyl ester; (i) 6-octadecenoic acid, methyl ester, (Z)-; (j) methyl stearate; (k) hexadecanoic acid, 2-hydroxy-1-(hydroxymethyl)ethyl ester; (l) octadecanoic acid, 2,3-dihydroxypropyl ester; (m) 13-docosenamide, (Z)-; (n) *N*,*N*’-methylenebis(oleamide), (Z, Z)-; (o) squalene; (p) α-tocopheryl acetate; (q) campesterol; (r) stigmasterol; (s) γ-sitosterol; (t) 4-campestene-3-one; (u) 9,19-cyclolanost-24-en-3-ol, acetate, (3.beta.); (v) 4,22-cholestadien-3-one; and (w) stigmast-4-en-3-one.
