# Supplementary material for: Phytochemicals from Leucas zeylanica Targeting Main Protease of SARS-CoV-2: Chemical Profiles, Molecular Docking, and Molecular Dynamics Simulations
Source: Biology (Basel). 2021 Aug 17;10(8):789. doi: 10.3390/biology10080789 (PMC8389631; doi:10.3390/biology10080789)
Supplement: Supplementary file 1 [file biology-10-00789-s001.zip › biology-1275291-supplementary.pdf]

Supporting information

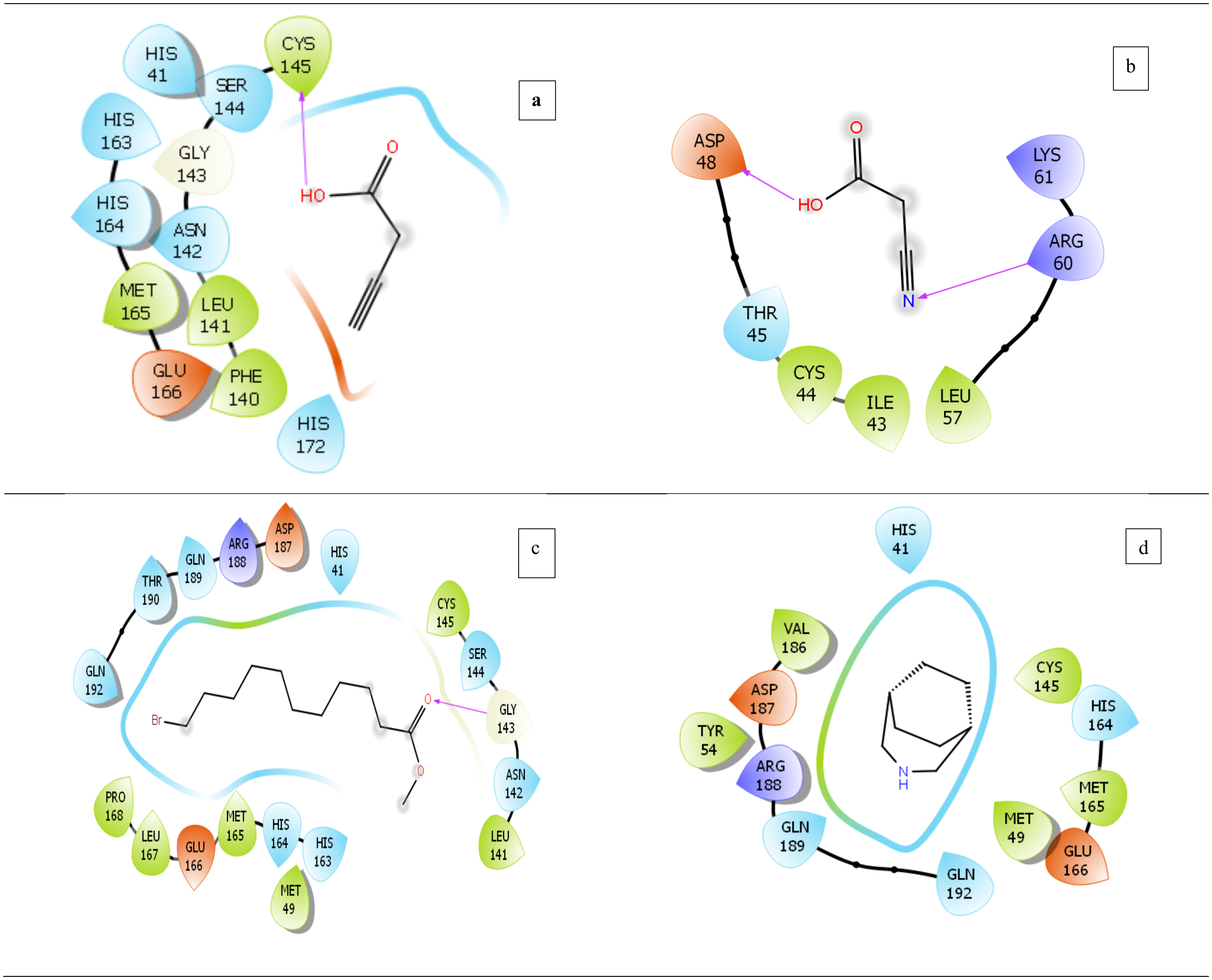

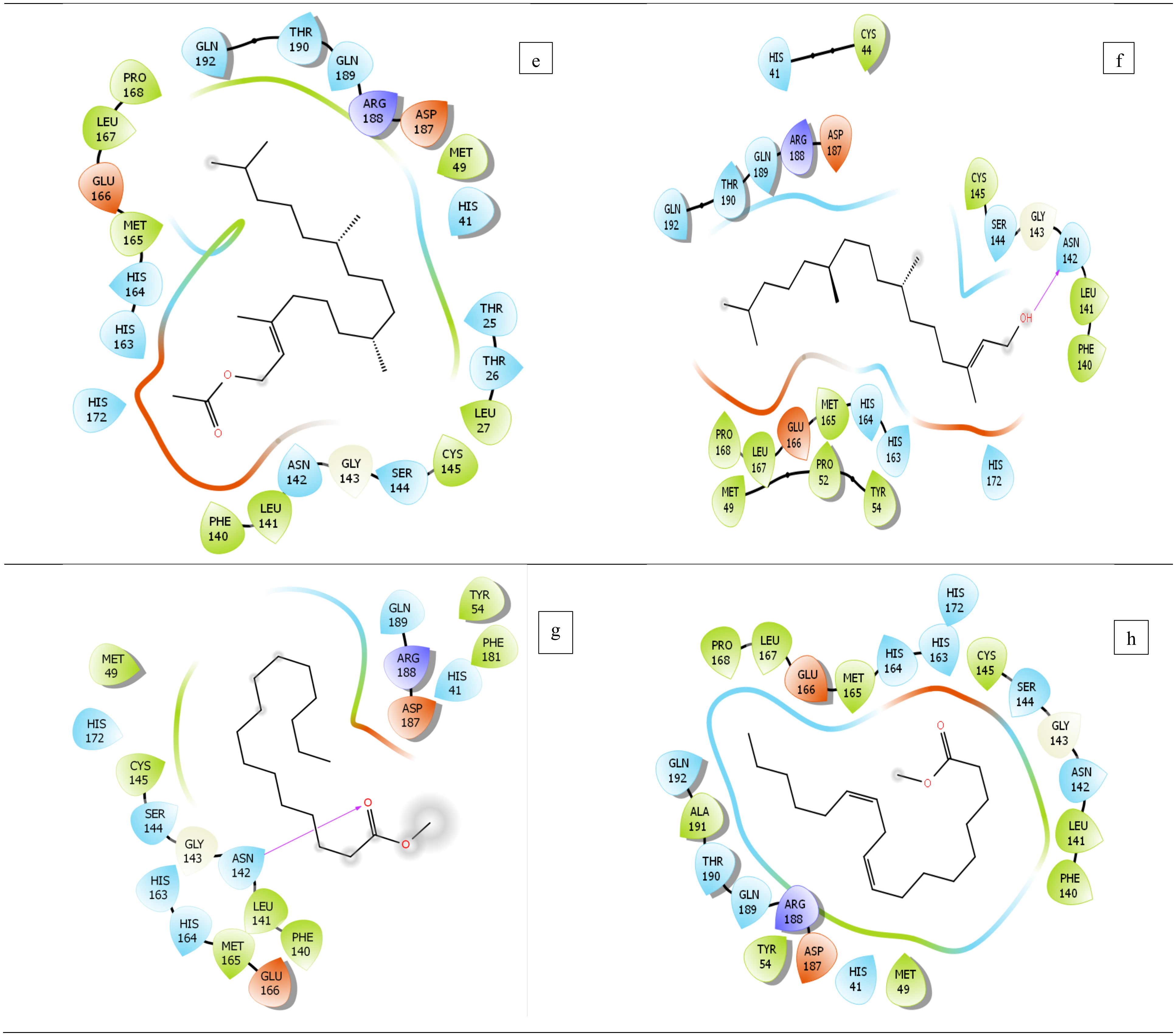
e
GLN 192
THR 190
GLN 189
ARG 188
ASP 187
MET 49
HIS 41
THR 25
THR 26
LEU 27
CYS 145
SER 144
GLY 143
ASN 142
LEU 141
PHE 140
HIS 172
HIS 163
HIS 164
MET 165
GLU 166
LEU 167
PRO 168
O
O
f
HIS 41
CYS 44
ASP 187
ARG 188
GLN 189
THR 190
GLN 192
CYS 145
SER 144
GLY 143
ASN 142
LEU 141
PHE 140
OH
MET 165
HIS 164
GLU 166
HIS 163
PRO 168
LEU 167
PRO 52
MET 49
TYR 54
HIS 172
g
GLN 189
TYR 54
PHE 181
ARG 188
HIS 41
ASP 187
MET 49
HIS 172
CYS 145
SER 144
GLY 143
ASN 142
HIS 163
LEU 141
PHE 140
HIS 164
MET 165
GLU 166
O
O
h
HIS 172
PRO 168
LEU 167
HIS 164
HIS 163
CYS 145
GLU 166
MET 165
SER 144
GLY 143
GLN 192
ASN 142
ALA 191
LEU 141
THR 190
GLN 189
ARG 188
PHE 140
TYR 54
ASP 187
HIS 41
MET 49
O
O

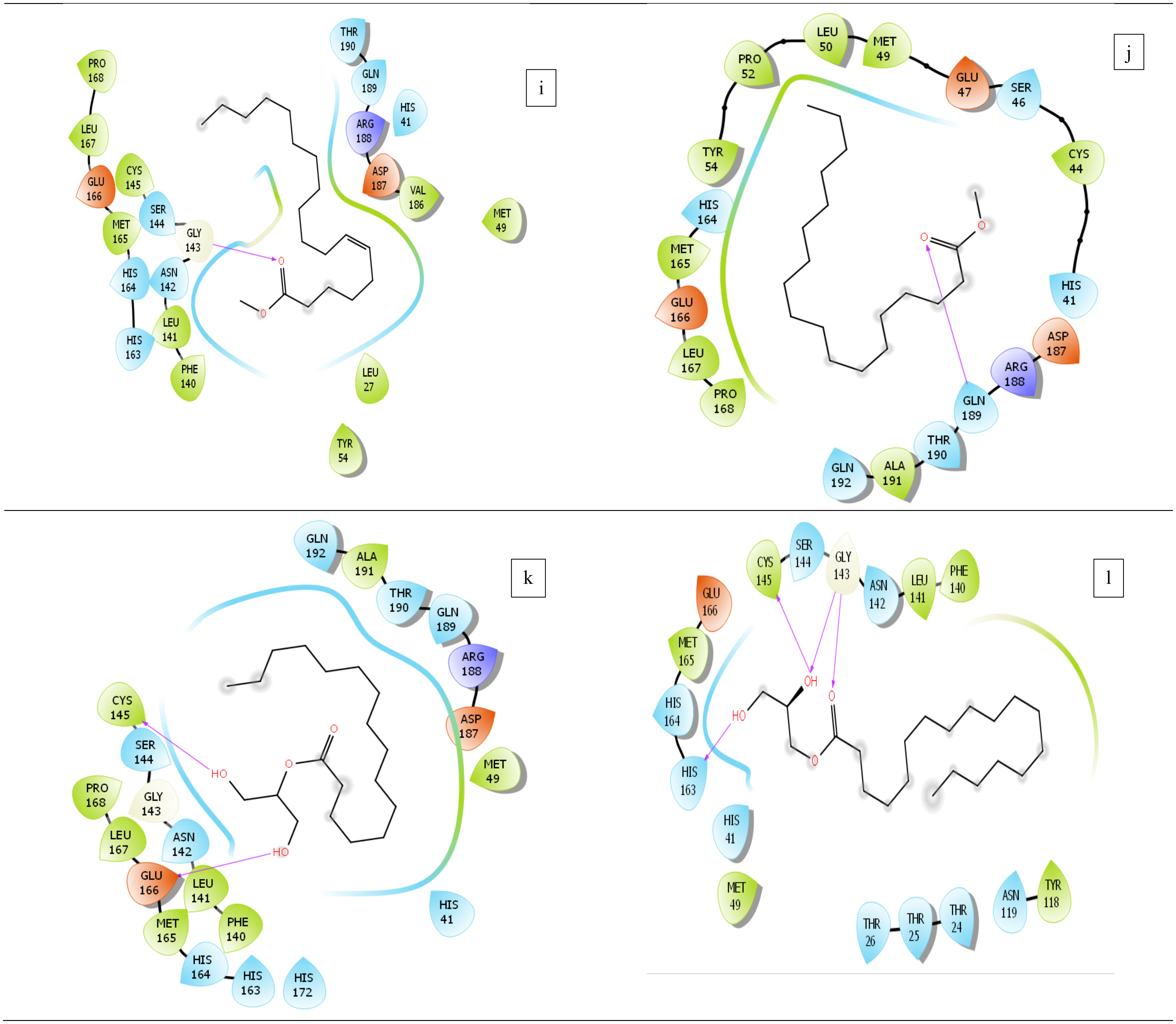
i
PRO 168
LEU 167
GLU 166
CYS 145
SER 144
MET 165
GLY 143
HIS 164
ASN 142
LEU 141
HIS 163
PHE 140
THR 190
GLN 189
HIS 41
ARG 188
ASP 187
VAL 186
MET 49
LEU 27
TYR 54
O
O
j
LEU 50
MET 49
PRO 52
GLU 47
SER 46
CYS 44
TYR 54
HIS 164
MET 165
GLU 166
LEU 167
PRO 168
HIS 41
ASP 187
ARG 188
GLN 189
THR 190
ALA 191
GLN 192
O
O
k
GLN 192
ALA 191
THR 190
GLN 189
ARG 188
ASP 187
MET 49
CYS 145
SER 144
PRO 168
GLY 143
LEU 167
ASN 142
GLU 166
LEU 141
MET 165
PHE 140
HIS 164
HIS 163
HIS 172
HIS 41
HO
HO
O
O
l
SER 144
GLY 143
CYS 145
ASN 142
LEU 141
PHE 140
GLU 166
MET 165
HIS 164
HIS 163
HIS 41
MET 49
THR 26
THR 25
THR 24
ASN 119
TYR 118
OH
HO
O
O

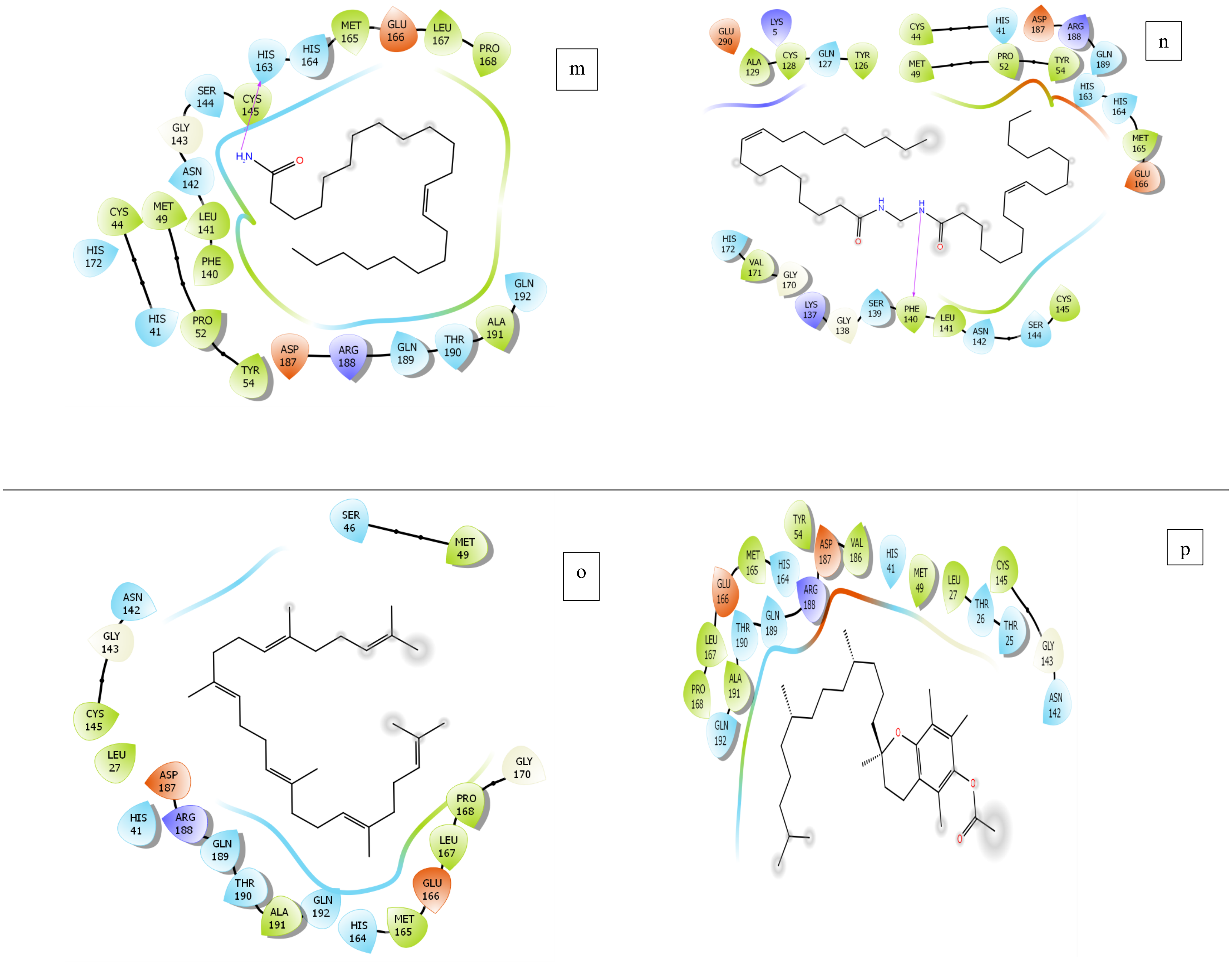
m
MET 165
GLU 166
LEU 167
PRO 168
HIS 164
HIS 163
SER 144
CYS 145
GLY 143
ASN 142
CYS 44
MET 49
LEU 141
HIS 172
PHE 140
HIS 41
PRO 52
TYR 54
ASP 187
ARG 188
GLN 189
THR 190
ALA 191
GLN 192
n
GLU 290
LYS 5
ALA 129
CYS 128
GLN 127
TYR 126
CYS 44
MET 49
HIS 41
ASP 187
ARG 188
PRO 52
TYR 54
GLN 189
HIS 163
HIS 164
MET 165
GLU 166
HIS 172
VAL 171
GLY 170
LYS 137
GLY 138
SER 139
PHE 140
LEU 141
ASN 142
SER 144
CYS 145
o
SER 46
MET 49
ASN 142
GLY 143
CYS 145
LEU 27
ASP 187
HIS 41
ARG 188
GLN 189
THR 190
ALA 191
GLN 192
HIS 164
MET 165
GLU 166
LEU 167
PRO 168
GLY 170
p
TYR 54
ASP 187
VAL 186
MET 165
HIS 164
HIS 41
MET 49
LEU 27
CYS 145
GLU 166
ARG 188
THR 26
THR 25
GLY 143
THR 190
GLN 189
LEU 167
ALA 191
PRO 168
GLN 192
ASN 142

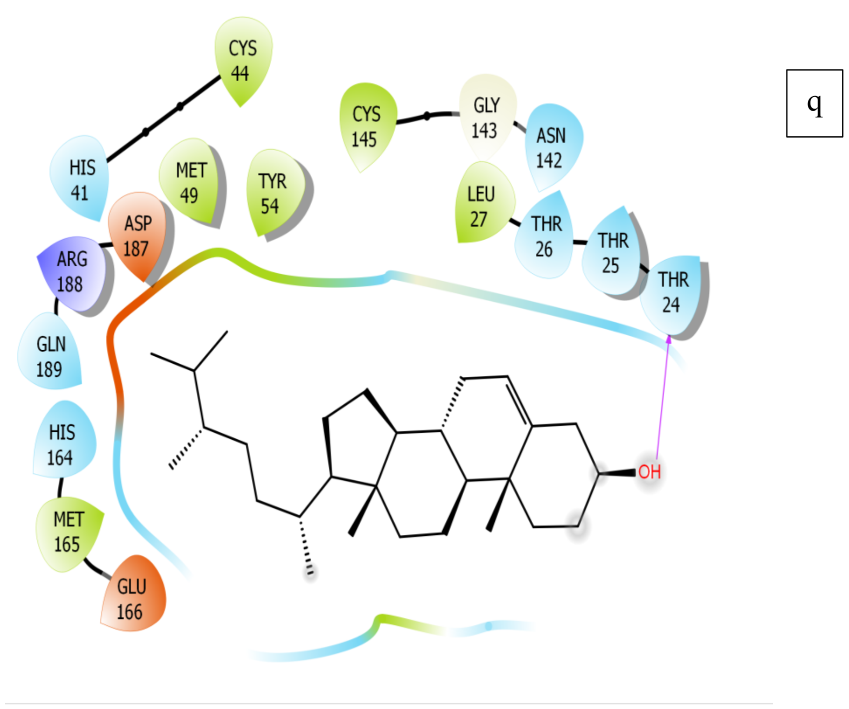
q
CYS 44
HIS 41
MET 49
TYR 54
CYS 145
GLY 143
ASN 142
LEU 27
THR 26
THR 25
THR 24
ASP 187
ARG 188
GLN 189
HIS 164
MET 165
GLU 166
OH

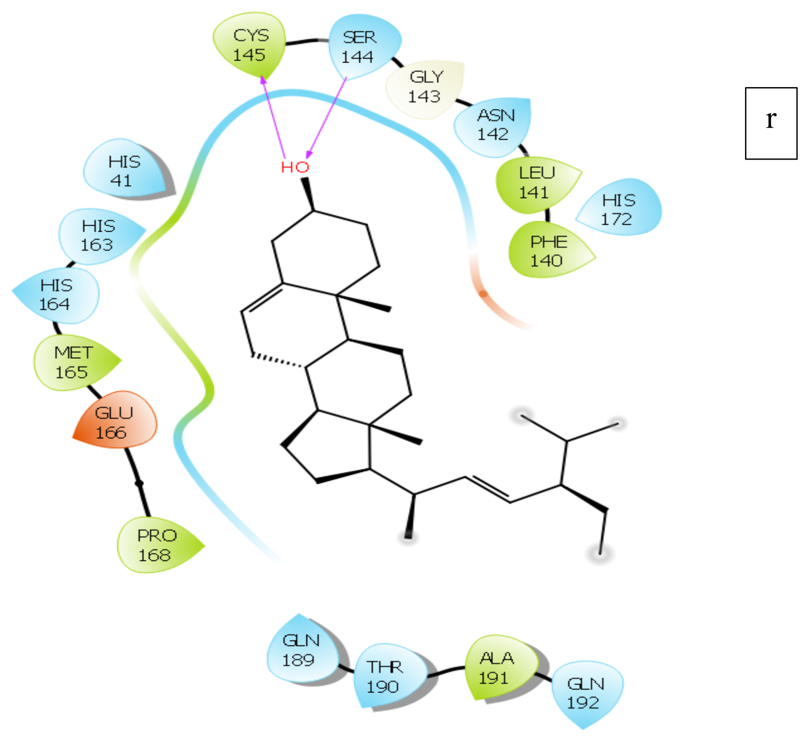
r
CYS 145
SER 144
GLY 143
ASN 142
LEU 141
HIS 172
PHE 140
HIS 41
HIS 163
HIS 164
MET 165
GLU 166
PRO 168
HO
GLN 189
THR 190
ALA 191
GLN 192

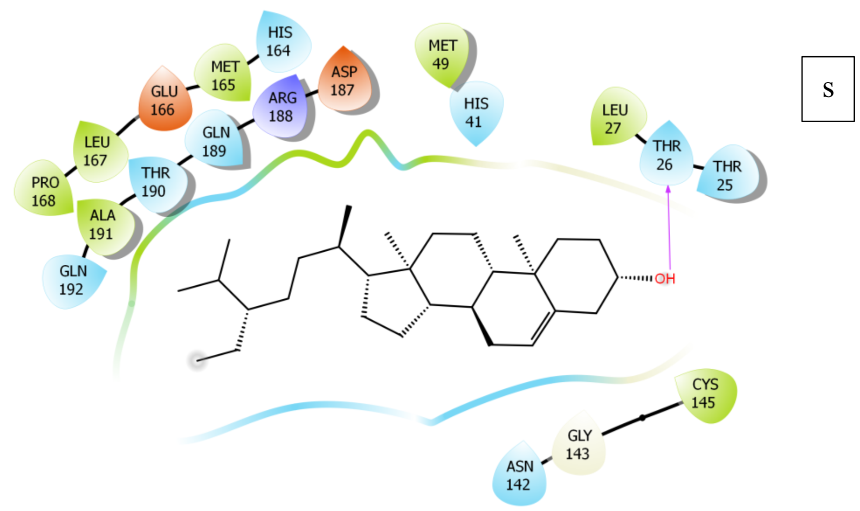
s
HIS 164
MET 165
GLU 166
ASP 187
ARG 188
GLN 189
MET 49
HIS 41
LEU 27
THR 26
THR 25
LEU 167
PRO 168
THR 190
ALA 191
GLN 192
OH
CYS 145
GLY 143
ASN 142

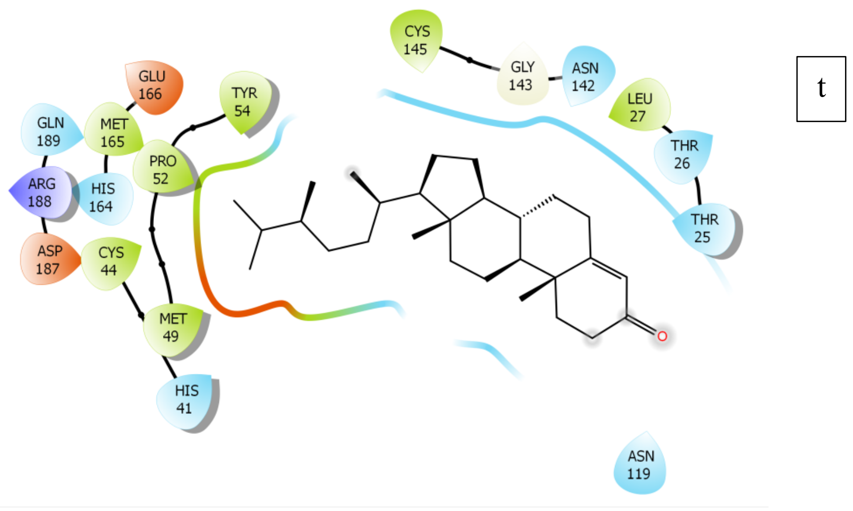
t
CYS 145
GLY 143
ASN 142
LEU 27
THR 26
THR 25
GLU 166
TYR 54
GLN 189
MET 165
PRO 52
ARG 188
HIS 164
ASP 187
CYS 44
MET 49
HIS 41
ASN 119
O

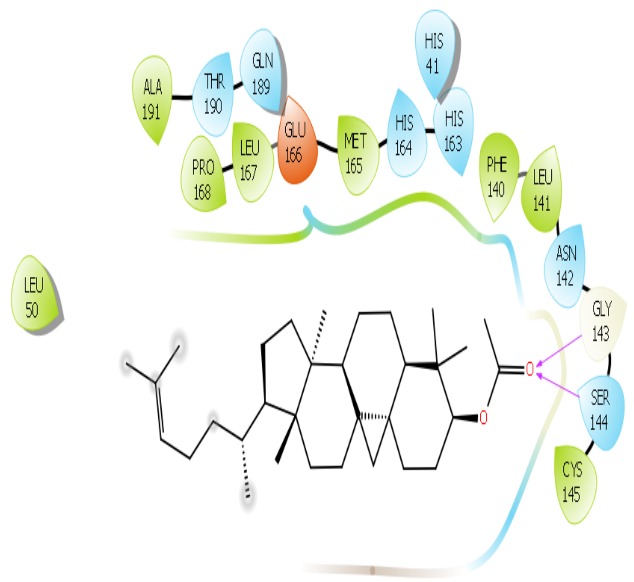

u

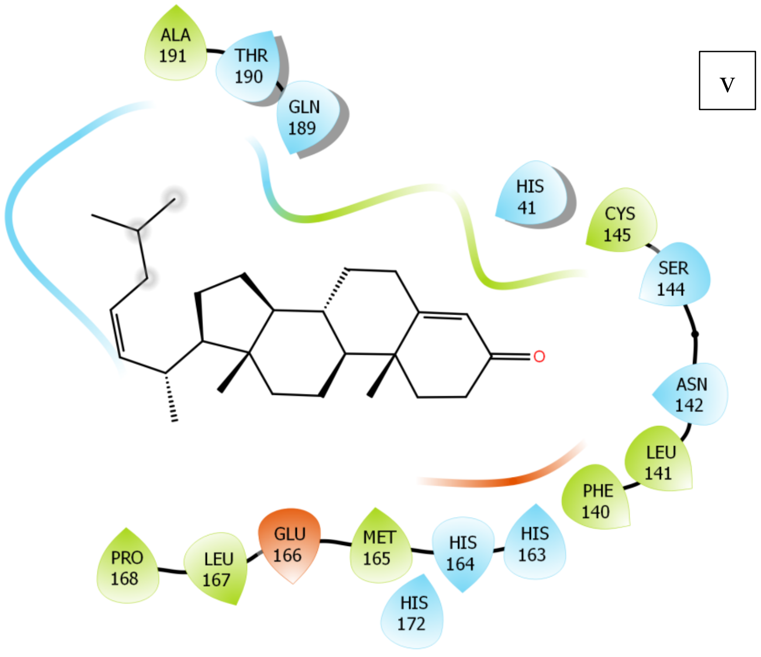

v

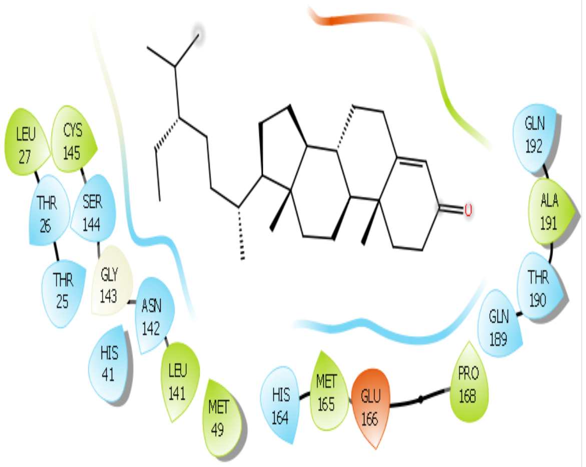

w

Figure S1: 2D representation of 23 compounds against the SARS-CoV-2 receptor (PDB: 6LU7). (a) 3-butynoic acid, (b) Acetic acid, cyano-, (c) Undecanoic acid, 11-bromo-, methyl ester, (d) 3-azabicyclo[3.2.2]nonane, (e) Phytol acetate, (f) 3,7,11,15-tetramethyl-2-hexadecen-1-ol, (g) Hexadecanoic acid, methyl ester, (h) 9,12-octadecadienoic acid (Z,Z)-, methyl ester, (i) 6-octadecenoic acid, methyl ester, (Z)-, (j) Methyl stearate, (k) Hexadecanoic acid, 2-hydroxy-1-(hydroxymethyl)ethyl ester, (l) Octadecanoic acid, 2,3-dihydroxypropyl ester, (m) 13-Docosenamide, (Z)-, (n) N,N'-Methylenebis(oleamide), (Z, Z)-, (o) Squalene, (p) alpha.-Tocopheryl acetate, (q) Campesterol, (r) Stigmasterol, (s) Gamma-sitosterol, (t) 4-campestene-3-one, (u) 9,19-cyclolanost-24-en-3-ol, acetate, (3.beta.), (v) 4,22-cholestadien-3-one, and (w) Stigmast-4-en-3-one.
